# *‘It’s been a lifelong thing for me’*: parents’ experiences of facilitating a healthy lifestyle for their children with severe obesity

**DOI:** 10.1186/s12889-023-15780-y

**Published:** 2023-06-19

**Authors:** Liz A. Saunders, Ben Jackson, Lisa Y. Gibson, Justine Doust, James A. Dimmock, Elizabeth A. Davis, Lyndsey Price, Timothy Budden

**Affiliations:** 1 School of Human Sciences (Exercise and Sport Science), The University of Western Australia, Perth, Australia; 2 Telethon Kids Institute, The University of Western Australia, Subiaco, WA Australia; 3 Paediatric Consultation Liaison Program, Child and Adolescent Mental Health Service, Perth Children’s Hospital, Western, Australia; 4 School of Medical and Health Sciences, Edith Cowan University, Perth, Western Australia; 5 Healthy Weight Service, Department of Endocrinology and Diabetes, Perth Children’s Hospital, Western, Australia; 6 Department of Psychology, College of Healthcare Sciences, James Cook University, Townsville, Australia

**Keywords:** Parental levels of obesity, Severe obesity, Fixed mindset, Obesity narrative, Weight stigma, Isolated and unsupported parents

## Abstract

**Objective:**

For parents and guardians, assisting children/adolescents with severe obesity to lose weight is often a key objective but a complex and difficult challenge. Our aim in this study was to explore parents’ (and guardians’) perspectives on the challenges they have faced in assisting their children/adolescents with severe obesity to lead a healthy lifestyle.

**Methods:**

Thirteen parents/guardians were interviewed from a pool of families who had been referred but *did not* engage between 2016 and 2018 (*N* = 103), with the Perth Children’s Hospital Healthy Weight Service, a clinical obesity program for children/adolescents (parent age M = 43.2 years, children age *M* = 10.3 years). Using semi-structured interviews and thematic analysis, we identified 3 broad themes.

**Results:**

Parental weight-related factors reflected parents’ own lifelong obesity narrative and its effect on their own and their families’ ability to live a healthy lifestyle. Perceived inevitability of obesity in their child reflected parents’ feelings that the obesity weight status of their children/adolescent was a persistent and overwhelming problem that felt ‘out of control’. Lastly, parents reported challenges getting medical help stemming from co-morbid medical diagnosis in their child/adolescent, and difficulties with medical professionals.

**Conclusion:**

This study demonstrates that parents face challenges in supporting healthy lifestyle for children/adolescents with severe obesity due to parents own internal weight biases and their negative experiences within the healthcare system when seeking help.

## Introduction

Severe obesity in children/adolescents is defined as as a body mass index (BMI) ≥ 120% of the 95th percentile for age and sex, or an absolute BMI ≥ 35 kg/m^2^[1]. Obesity in childhood consequences are many and predominantly negative. Obesity in childhood has clear associations with adverse physical health outcomes [2–4], and there is compelling evidence that obesity in childhood is predictive of obesity in adulthood [2, 3, 5]. Social consequences of obesity include bullying [5–7], discrimination [8], perceived external negative perceptions [9, 10], and social stigma [11]. Psychological consequences can include low self-esteem and disturbances in body image that often persist into adulthood [5, 12]. Long-term outcomes for children/adolescents with *severe* obesity are especially poor, with some studies highlighting that severe obesity in childhood increases the risk of premature adult mortality, independent of current adult BMI [13]. However, in relation to treating severe obesity, the notion of calories in, calories out is an oversimplified solution to a complex disease [10] and does not acknowledge that eating well and engaging in physical activity is a recommendation for *all* people regardless of their health or illness status. What is not yet clear, however, is the range of barriers and challenges that parents experience when attempting to support a healthy lifestyle for their child/adolescent with severe obesity. Children/adolescents with severe obesity and their families are a critically important but often underrepresented population in paediatric health research as these families are often hard to access [14], especially when families are *not engaged* in treatment programs [15], and thus are not as well studied as ‘non-clinical’ populations (i.e., those without severe obesity).

Parents (and guardians) can be especially influential in affecting the weight status of children/adolescents. As well as exerting uncontrollable influence through genetic, biological and epigenetic factors [2, 16], parents can indirectly influence children/adolescents by acting as role models, and can directly facilitate change by influencing family health behaviours and choices [17]. There is evidence in the obesity literature related to children/adolescents that obesogenic contextual lifestyle behaviours (i.e., unhealthy diets, physical inactivity, sedentary behaviours) of parents may lead to children/adolescents engaging in these same behaviours [18]. However, it’s unclear what effect these factors have on—and how they are managed —in families with a child/adolescent with *severe* obesity.

Obesity in childhood treatment most commonly takes the form of lifestyle modification programs and it is generally acknowledged that a parent-led, family-based approach involving both parent and child/adolescent is the most effective for managing or reducing the child/adolescent’s level of obesity [19]. Although child/adolescent weight loss is not always the primary focus of interventions (i.e., programs often focus on healthy behaviours and relationships with exercise, food choices, and body positivity), at the level of severe obesity, reducing a child/adolescent’s level of obesity is an important health goal. Optimal program structure combines parent-led family behaviour modification (such as parenting skills) and educational topics, such as healthy nutrition, appropriate exercise guidelines, and a formal program of exercise for the child/adolescent [20, 21]. For children/adolescents with severe obesity, treatment is typically delivered in tertiary hospital-based settings, i.e. a clinical program, involving multidisciplinary specialists to address various problematic and maladaptive aspects of the obesity and to manage medical comorbidities [22]. However, although these lifestyle modification family-based programs are the cornerstone of clinical treatment for severe obesity in children/adolescents, it seems that many of these programs do not elicit immediate or long-term weight loss outcomes [22, 23]. To date, much of the research on reasons for unsuccessful clinical program outcomes has focussed on parents reported reasons for attrition from clinical programs [24, 25], the experience within a clinical program itself [26], or evaluating the outcomes of pilot or new types of programs [27]. Although there is well-developed literature documenting the difficulties that parents face supporting children/adolescents with lower levels of overweight or obesity [14, 28, 29], relatively little research has been undertaken on the *broad challenges that parents face in supporting weight loss and lifestyle behaviours* among children/adolescents with *severe* obesity. In addition, much of the research on severe obesity in children/adolescents takes a *retrospective* approach, i.e., focused on families that have actively sought treatment for their child/adolescent’s severe obesity. As such, among these families, less is known regarding the *everyday challenges* faced by those parents *who have not or choose not* to engage with a clinical tertiary program. Guided by the gap in the literature outlined above, the purpose of the current study was to solicit insight into the experiences of this hard-to-access group using a qualitative approach. Specifically, we sought to better understand the experiences and challenges of parents *who were referred but did not engage* in a tertiary clinical intervention program, in *facilitating a healthy lifestyle* for their child/adolescent with severe obesity.

## Method

### Philosophical perspective

This study was guided by an interpretivist paradigm, underpinned by a relativist ontology (i.e. the notion that there are multiple ‘realities’), and a subjectivist epistemology (i.e. one’s life experiences shapes their perception of the world) [30]. Within this paradigm, it is understood that knowledge is co-created between participants and the researcher(s), and researchers focus on finding meaning within their own and (where possible) participants’ historical, temporal, cultural, and subjective circumstances. A reflexive approach was also adopted throughout the study. Reflexivity requires an acknowledgment that researchers’ perspectives may influence the research process and influence their research findings [31]. With this in mind, the author team had research experience in the area of obesity [32, 33] as well as programming expertise in clinical and community settings, and some were also parents. The lead author had a longstanding relationship with the clinical obesity intervention program from which participants were recruited—this relationship allowed access to a hard-to-reach clinical population and meant that the lead author held knowledge about the structure of programs, the staff within the program, and the broad experiences of participants referred to the program, and a desire to help these families share their experiences.

### Participants and procedure

Ethical approval to conduct this study was obtained from the Child and Adolescent Health Services Human Research Committee (CAHS HRC) and the University of Western Australia Human Research Ethics Committee. The Healthy Weight Service (HWS), based in Perth, Western Australia, is a 12-month, multidisciplinary, psycho-educational, family lifestyle intervention program. Participation in the HWS is voluntary and costs are covered by the Australian Government Medicare Scheme. Eligibility criteria for the HWS include a BMI*-z* score > 97th percentile; diagnosed pre-diabetes (i.e., an impaired fasting glucose and impaired glucose tolerance), or BMI-*z* score > 95th percentile and two co-morbidities (i.e. high blood pressure, depression, or obesity-related joint or bone problems). Participants were recruited using criterion-based and maximum variation sampling methods [34]. The criteria for inclusion were: A) parents (both Mother and Father or primary caregiver) of any family that had been referred to the HWS from 2016 to 2018, B) parents of families living in the Perth metropolitan area, C) parents of children/adolescents aged under 18 years, and D) parents from families *who had not undertaken* a HWS program prior to their interview date (i.e., had no experience of this clinical obesity program for children/adolescents). This sampling approach intended to capture varying socioeconomic backgrounds due to the wide inclusion criteria and because the recruitment program represented the only clinical obesity speciality treatment centre for children/adolescents with severe obesity in Western Australia.

Eligible parents were mailed an invitation letter outlining the purpose and nature of the study. The first author subsequently contacted all parents or primary caregiver by phone (with the exception of those who returned a written response declining to participate) to gauge interest in participating. Parents/primary caregivers of eligible families elected to participate in the study. Participants provided written informed consent prior to their participation in their interview. Participants also provided consent for the lead author to access anthropometric data relevant to the child/adolescent (i.e., referral weight and height) as per Ethics approval. All contact and outcomes were recorded (e.g., interview times and dates).

In total, 103 families were contacted between October 2017 and May 2018 either by return mail or through phone contact with the first author. A final sample of 13 families were interviewed. The sample included eleven parent/caregiver-only interviews with one parent/caregiver from each family, and one interview with parents from two separate families. Participant demographics are listed in Table 1. Parents BMI’s ranged from 23.5 to 52.4 (*M* = 36.4, *SD* = 10.20), with two parents in the ‘normal weight’ range, one ‘overweight’, and 10 classified as ‘obese’ according to their BMI. Parents were aged 36 to 50 years (*M* = 43.2, *SD* = 4.63) and had children/adolescents aged 6.8 years to 17.1 years at the time of interview (*M* = 10.34, *SD* = 3.49). Eight children/adolescents were male and five were female. Two parents interviewed were male and the remaining 11 female. Seven families were intact (parents of the child/adolescent were still in a relationship), two families were separated from the other parent but with new partners, three families were separated from the other parent and the parent was single, and one person interviewed was a grandmother with primary custody of the child/adolescent. Interviews lasted from 42 min to 1 h and 57 min (*M*_=_ 105 min, *SD* = 62.85).

**Table 1:** Family Demographic Factors

Factor	*n*	Mean (*SD)*	Range
Child/Adolescent Referral BMI-z	13	2.61 (0.43)	1.85–3.19
Child/Adolescent BMI-z at interview	11	2.48 (0.47)	1.36–3.06
Child/Adolescent age at referral (years)	13	9.33 (3.48)	4.6–15.3
Child/Adolescent age at interview (years)	13	10.34 (3.57)	6.4–17.1
PCG BMI	11	36.33 (10.04)	23.5–52.4
PCG Age (years)	5	43.2 (4.63)	36–50
SEIFA Ranking WA	13	5 (3.11)	1–10

*Note. N* = 13; *BMI range* (kg/m^2^)-Normal weight (18.5–24.9), Overweight (25-29.9), Obesity Class I (30-34.9), Obese class II (35-39.9), Obesity class III SEVERE (≥ 40); *PCG =* Patient Care Giver; Socio-Economic Indexes for Areas *(*SEIFA) ranking range from 1 = most disadvantaged to 10 = Least disadvantaged

### Data collection

A semi-structured interview guide was developed by the authors, all of whom are experienced in conducting semi-structured interviews and familiar with relevant psychology and obesity literature. All questions were structured in an open-ended format, with follow-up probing questions used for elaboration and clarification to glean as much information from participants as possible [34]. All interviews were conducted by the first author, who was responsible for audio-recording and taking written notes on each discussion [34]. All families who participated in this study were unknown to the lead author. At the conclusion of the interviews, participants self-reported their own and their child’s current height and weight.

Data collection was guided by the principle of pragmatic saturation. Data saturation is typically considered the point where information obtained from participants becomes repetitive, and thus conducting further interviews is unlikely to yield new information [34]. This work does not (and cannot) claim to represent the views of *all* parents referred to a clinical obesity intervention program for children/adolescents with severe obesity; it’s entirely plausible that further themes may have emerged if additional interviews had been conducted. However, a pragmatic perspective to saturation was adopted—data collection was ceased at the point at which, following discussion with all co-authors, a sufficiently detailed account of parents’ experiences had been obtained.

### Data analysis

Audio recordings were reviewed by the first author and transcribed verbatim. Data were analysed using a reflexive thematic analysis process that was assisted by computer analysis data guidelines [34]. Analyses were conducted using the following steps: familiarisation with data through immersion by the lead researcher listening to audio recordings of interviews and reading transcripts; generating initial codes involving identifying and labelling common themes using NVivo qualitative data analysis Software (QSR International Pty Ltd); ordering data within transcripts into meaning units and categorising these meaning units into themes; and assigning emergent code/theme names. Themes were constantly reviewed, consisting of revising extracts and considering whether clear patterns had formed within each theme. Original transcripts were cross-checked to ensure all themes were represented, using a constant comparison method to ensure meaning units were reflective of identified themes. Themes were then named and defined allowing the identification of sub-themes (authors LS, J.A.D and BJ). An initial thematic framework was identified including both themes of a semantic (i.e., expressly described) and latent (i.e., meaning was ‘underneath’ what was expressly stated) nature. Analysis involved a series of ‘critical friends’ meetings (i.e., with either two or three co-authors familiar with thematic analysis techniques) to discuss the initial framework, meaning units, themes, and interpretation (authors LS, TB, J.A.D and BJ). This process encouraged reflection and exploration of alternative meanings and interpretations [35]. Through this process, themes were renamed, reordered, some themes were condensed, and some completely discarded as they did not fit the scope of the project or research question (i.e., the challenges facing parents with severe levels of obesity in assisting their child/adolescent with severe obesity to live a healthy lifestyle). Lastly, the report was written, allowing for additional interpretation of data and consolidation of final themes.

## Results

The aim of this study was to better understand the experiences and challenges of parents/caregivers who were not or had chosen not to engage in a clinical tertiary obesity intervention program for children/adolescents with severe obesity, in facilitating a healthy lifestyle for their child/adolescent with severe obesity. Three major themes were identified—parents’ own lifelong obesity narrative, parents’ perceived inevitability of child/adolescent levels of obesity and parents’ perceived challenges in getting medical help for child/adolescent reduction in levels of obesity. Themes, sub-themes, and exemplar meaning units are displayed in Tables 2 and 3, and 4 and described in detail below.

**Table 2:** Themes, sub-themes and exemplar meaning units reflecting Parental own weight-related factors

Theme	Sub-theme	Exemplar meaning unit
Parent lifelong obesity narrative	Parent family history of weight-related issues influencing in the present	(P7) I grew up with knowing a lot of this stuff. My Mum and my Step-Dad were mega health freaks sort of thing. So, I know all this, but throughout my life there have been things that have brought on depression. That is where I struggle. I know all the healthy things and exercise with growing up with it. (P4) Growing up [Mum] was always doing things. She was doing the rebound exercising, the trampoline in the ‘70s and then at the same time she’d be like, we need to clean our systems out and she’d do this chicory soup thing which was literally lettuce in boiling water in a bowl with a bit of olive oil on the top and salt and that was dinner with a bit of brown bread. We all had to do it….we hated it.
	Weight out of control, not knowing how to get started	(P12) I’m not going to try anymore. I get a bit defeated and yeah - I don’t want to. (P2) I’ve been putting it on hold too going “You know, I can’t be bothered, I haven’t got time”, but I’ve got 30 kilos, or nearly 40 kilos to lose myself to be back to my healthy, what I used to be. I think I’m using – it’s not an excuse, but I keep saying “Next year when I finish I’m going to have more time”. But I feel like I’ve just put my life on hold now for three years. (P4) I haven’t really stuck to anything. So I don’t even know if I can say that I’ve tried.
	Parent failed weight-loss attempts impact	(P12) The soup diet and was doing Jenny Craig for a couple of months when I was younger, Herbalife at one stage when I was about 20, but that wasn’t very long you know. I’m a shocker you know, I give it a go for a month or a week and that’s it you know. (P4) I have actually never succeeded on a diet. The only diet that worked for me was when his dad and I split up and he hooked up with a ballerina after me and I lost my appetite. (P2) I was so desperate to make that change to lose that weight again, and go “You know what, I’m sick of being…that was after babies, yeah. Not after my last one, that was after [child name], yeah. And I haven’t been back since. I basically just got disheartened, then I went back to my studies full time, so that stopped everything,

**Table 3:** Themes, sub-themes and exemplar meaning units reflecting Parent perceived inevitability of child with obesity

Theme	Sub-theme	Exemplar meaning unit
Parent perceived inevitability of child with obesity	Want to help and fault of genetics	(P6) At the end of the day I will do anything for him. I do want him to be healthier and I could see his weight was gaining. (P12) You know that Danny De Vito and Arnold Schwarzenegger movie Twins, well [child name] is Danny De Vito, he got all the shit. (P9) I think it’s a lot of genetics as well…We all do (have an issue with any weight or shape), that’s why I think it’s a lot of genetics as well.
	Acknowledgement of the gravity of problem and worry about child future	(P3) I didn’t want him to be self-conscious about it or ashamed or any of that sort of stuff because it’s going to be part of his life and I don’t want him sort of body-shaming and all that sort of stuff. (P5) [Child name] has obviously got the big issue. We just tell him we don’t want him to be like us…Battle of the bulge. We don’t want him to grow that way. We actually said to him, we don’t want any cause for bullying, we don’t want that extra pressure for him.
	Frustration about their child’s level of obesity	(P3) Theres lots of things that we miss out on and there’s lots of restrictions in life and I think it’s always having to be the fun police. (P12) They want people to be individual but everybody has still got to stick to the norm, you know, you’ve still got to be, it just shits me sometimes.

**Table 4:** Themes, sub-themes and exemplar meaning units reflecting Parent perceived challenges in getting medical help for child weight loss

Theme	Sub-theme	Exemplar meaning unit
Child comorbid medical diagnosis makes it hard to achieve any child weight loss	Attribution of level of obesity to medical diagnoses	(P6) He’s got low muscle tone, he doesn’t really retain a lot. (P4) We struggle because [Child name], with his autism, he’s come a long way with his food now but when he was younger he was very picky and fussy about food, to the point that if he was made to eat something he didn’t like he would literally throw up at the table. (P3) I think because we have been doing everything we can [to reduce child’s obesity], and given [child’s brain condition diagnosis] condition, it’s just something I can’t seem to get on top of.
Difficulties with Medical professionals in seeking support and advice for child weight loss	Repeated efforts to get help from Primary care medical professionals - GPS	(P5) The GP just said no, it’s okay. We kept going back to different GPs. (P2) We want to help her, because she’s been seeing the paediatrician for two years, but nothing changed. (P8) The doctor wouldn’t give me one [referral]. He said “You don’t need it. What do you need it for?”. And I said “Look at this child”. And so that’s when he said ‘No, we’ll just test the thyroid’.
	Frustration with the negative and unhelpful attitudes of specialists	(P3) I will try anything for the better and this is where I got a bit frustrated with [Child Psychologist]. (P4) I would guarantee that there’s a lot of kids on the spectrum that have weight issues because they have sensory issues and issues with persisting unless they’re interested. (P5) We went to Doctor [Specialist number 2] because we hadn’t heard from anyone at [weight loss program name], so we were sick of waiting. We went to Doctor [specialist number 1] first, then we went to Doctor [Specialist number 2].
	Loss of confidence in specialists’ advice	(P5) We went and saw the dietician and he [child] actually got upset at that appointment, but any information that I’ve gotten has been either really obvious information that everybody should know or it’s completely unreasonable, like instead of having fizzy drink buy soda water, make carrot juice and put soda water in carrot juice. (P8) I find them quite dismissive, they just don’t want to listen to what you’ve got to say. (P13) The dietitian’s advice was not very helpful. Well, just, you know, it was common sense.

### Parent lifelong obesity narrative

In describing challenges associated with helping their child/adolescent live a healthy lifestyle, many parents described their own history of lifelong issues or challenges with weight and shape. This history included obesity in childhood and bullying, parents’ levels of obesity being ‘out of control’, children/adolescents being aware of their parents’ struggle with their own weight, perceived cyclical failed attempts at weight loss, feelings of sadness and self-defeating evaluation, and acknowledging the importance of, but avoiding engagement in, health-related behaviours. The parents who reported previous attempts at weight loss all had levels of overweight or obesity at the time of interview.

The negative narrative of obesity was long-standing and entrenched for many parents. In describing challenges in supporting their child/adolescent’s healthy lifestyle, parents spoke of their own experiences of childhood and their parents’ negative weight-related behaviours, and how this impacted on their own health behaviour beliefs for their own children, such as Mel (P4):

I think it made us obsessed about food because there was just no flavour and no texture, and it was just quite miserable. Well, I think that we all—we’re emotional eaters and I can’t plan… so if something good happens then we celebrate with a treat. If something bad happens we commiserate with a treat and [sister name] and I, both of us have talked about this.

Parents commonly reported various negative weight-related experiences such as obesity in childhood and a history of weight-related teasing: “[My husband] was bullied for his weight when he was younger. But like us, his Mum took him to the doctor, and they just said, you’ll grow, it’s fine. It’s not so fine” (Robyn, P5).

Parents also described their feelings about their own current weight, their attitude towards making health related behaviour change, and often the effect this had on their child/adolescent. Parents reported their own weight as having ‘got out of hand’ or not knowing how to get started with weight loss. As Sharon (P2) noted, “And then when they put you on the scales and you’re like “Have I put on 10 more kilos since Christmas? Oh my God. It’s like it’s not happening then”. Parents spoke about knowing their children had awareness of their (the parent’s) own struggle with their level of obesity. For instance, Robyn (P5) described, “I go to the gym six times a week, and the boys know that”, highlighting her children’s awareness of their parents’ behaviour, and their knowledge that she is perpetually aware of her weight status. Parents often spoke about feelings of sadness and self-critical evaluation of their current weight status, such as Karen (P7); “I’m slowly getting more and more overweight and older and increasing my chances of diabetes and stuff. Knowing that I have been fit and healthier in the past and remembering those times”, with several parents actively labelling themselves as lazy such as Chrissy (P9), “We’re all lazy”. Some parents, like Alexis (P12), described knowing their weight was a problem but were not actively interested in doing anything to change this “Everything is just emotional. That’s it and sometimes it’s just too hard”. Many parents also described examples of knowing they should, but avoiding engaging in, health-related behaviours with their children and family, like Sharon (P2):

With [child name] trying to say “Mum, let’s go walk, let’s go walk”, and I feel like I’m letting her down, because she’s trying so hard to make changes, and going “I want to walk, I want to walk, but I’m like, yeah, “Next week darling, I really can’t focus right now, I’m sorry, leave me alone, I’m on the computer”.

Parents described their own history of multiple and repeated attempts at weight loss—attending the gym, restrictive fad diets or dieting, weight loss supplements or pills, weight loss programs, health professional advice, and two parents had undergone or were planning bariatric surgery. Many parents spoke of a perceived failure of weight loss as their reason for stopping any health-related behaviour change:

I went to the gym for a year I was toning, so that was the good thing about it, but I wasn’t losing anything, and I wanted to go and lose weight. So, I lost a kilo in six months, and I had three days a week a personal trainer, I was going to aerobics classes, I was trying so desperately. Sharon (P2).

This narrative of perceived failure of weight loss was described in a cyclical nature—that is, parents tried a health-related behaviour change, perceived failure, stopped the newly adopted behaviour, and returned to their previous unhealthy lifestyle. This cycle was often described as negatively influencing parents’ own current motivation, where parents described the capacity to change as being too late or too hard, like Alexis (P12):

I was walking like an hour a day for five days a week and didn’t lose anything. It just deflates me and now it’s just even worse now because I’m not doing anything, that’s my fault. I’ve got my own issues.

Parents described examples of acknowledging, but not acting on, their own and their child/adolescent’s level of obesity in instances requiring them to also engage in health behaviours, like Sharon (P2): “They’re [all the kids] always punishing me, because I didn’t do it. They’re always saying “You promised me and we haven’t gone walking”. Nearly all the parents with obesity described instances of unhealthy family eating behaviours, often explained through a lens of rationalising these unhealthy options, such as Karen (P7); “We used to have cooked meals like a few years ago. Especially when our fridge died, and I couldn’t afford to get another fridge, that is when we started with the frozen meals. Convenience and money”. Parents also described how their lifetime of obesity had a negative effect on their attempts at health-related behaviour change their child’s current levels of obesity, like Karen (P7),

He [child/adolescent] knows about the healthier options because he’s had them in the past, just my motivation and energy. Yes, and just the confidence because I haven’t really learnt the skills throughout my life to do it and stuff as well.

In summary, it appeared that in this theme parents described a negative and pervasive lifelong obesity narrative which had a detrimental effect on their child, children, and/or family, and that parents felt was unlikely to change.

### Perceived inevitability of child with obesity

In discussing challenges experienced when supporting their child/adolescent to engage in a healthy lifestyle, parents felt a level of obesity was ‘inevitable’ for their child/adolescent, and that it presented an overwhelming long-term challenge no matter how hard parents tried to change it. At the time of interview, all but one child/adolescent had BMI-z scores (standardized score for gender and age) at or above the 99th percentile. All parents reported previous failed attempts at reducing their child’s level of obesity. Parents described wanting to help but that their motivation had been undermined by a sense of inevitability and hopelessness, feeling ineffectual due to past failures of reducing their child/adolescents level of obesity, ‘bad genes’, feelings of desperation due to past failures at reduction in obesity level, the gravity of their child’s severe obesity, feelings that their child/adolescents level of obesity was out of control, worry about their child’s long-term future, and child awareness of the gravity of the severity of obesity, frustration at the effort with no success, unwillingness to restrict child/adolescent diet, and defense of inactivity.

Although parents expressed a desire to help their child/adolescent, it seemed that parents’ motivation had been eroded or undermined by a sense of inevitability, hopelessness, and feelings of desperation for success in reducing their child\adolescents level of obesity, such as Carly (P3); “I’d do anything to learn more or help more”. Many parents attributed both their child’s severe obesity and often their own to “bad genes”, such as Chrissy (P9): “She’s built like her dad so she’s short and stumpy. She’s got a big belly. She’s got, yeah, unfortunately she’s got the bad genes”. Other parents described these ‘bad genes’ as limiting their capacity to change the child’s trajectory of severe obesity and describing it as being out of their control. Mel (P4), for example, noted: “I’m not sporty. [Dad’s] not sporty. We’re musos. It’s not even in his genes and he hasn’t been - unfortunately we couldn’t encourage him to do something that we don’t know how to do”.

All parents acknowledged the gravity of the severe level of obesity for their child and worried about their child’s long-term future, and described feeling sad for their child/adolescent due to their current severe obesity, such as Delia (P8): “I feel sad for her, because I think “Oh God, you’re only six, what are you going to be like when you’re 15, 20, if we don’t get on top of this?”. Many parents also perceived that their child was aware of the gravity of their severe obesity, such as Sharon (P2): “We want to help her, because she’s been seeing the paediatrician for two years, but nothing changed and at the last visit her weight went right up, and I saw her face change, and I thought ‘She’s not happy’.

Parents expressed strong feelings of frustration about their child’s level of obesity, which was expressed in several ways. Parents described the negative impact placed on themselves by the constant awareness and time focused on the severe obesity, such as Carly (P3): “I think it’s also very taxing on my husband and I”. This feeling of frustration also resulted in parent inaction which was counterproductive to goals of reducing their child/adolescent’s level of obesity. For instance, parents described an unwillingness to place dietary restrictions on their child as they wanted their child to just be ‘normal child’, such as Robyn (P5): “I just want him to be a normal kid. He’s going to get enough structure in his life, so I just think – I don’t want to have to say, oh, no mate”. Lastly, parents also described this frustration in a defensive manner promoting active rejection of any health-related change, even when faced with serious consequences for their family, such as Alexis (P12):

We’re all overweight,. And school is tough. My husband gets a bit edgy, not anti, but just…Yeah, like when Department of Children Services – they are going to take him off us [for child obesity level]- we were trying to do as best as we can, you know.

### Challenges getting medical help for reducing child/adolescent level of obesity

All parents described seeking help in various forms from medical professionals to help their child/adolescents level of obesity. Parents described difficulties with child comorbid medical diagnoses which made it difficult to achieve any reduction in child levels of obesity, and problems in seeking support and guidance from medical professionals.

#### Child comorbid medical diagnoses make it hard to reduce level of obesity

Nearly all parents discussed a chronic medical condition for their child/adolescent as an additional layer of complexity alongside their child’s severe obesity. Children/adolescents health conditions/diagnoses included, respiratory conditions (asthma, allergic rhinitis, sleep apnoea, ear nose and throat issues), neurological abnormalities (absent posterior bright spot), muscle and joint conditions (low muscle tone, join pain), mental health diagnosis (autism, attention-deficit/hyperactivity disorder, anxiety, depression), and metabolic conditions (fatty liver disease, high blood pressure). Many parents directly attributed their child’s severe obesity to their child’s comorbid medical diagnoses, and often minimised the role of unhealthy foods and behaviours, such as Sharon (P2):

I think it’s her asthma medications in the past, I think it’s a lot to do with her health that’s been unhealthy, her asthma, her allergic rhinitis, it’s constant, her hay fever that’s constant, probably her sleep apnoea. So, I don’t think it’s totally just been her diet, I think it’s, because she was active and fit, and she does her school stuff, sport and things. And she’s not one to say “No, I don’t want to go walking”.

Parents described wanting to support their child/adolescent in living a healthy lifestyle but being unsure how best to engage in health-related behaviour change that would be appropriate for their child’s medical diagnoses, such as Doug (P1):

Because with his asthma and everything – I was not an asthmatic kid so I didn’t have a problem. [Mum name] was not an asthmatic kid. Both of us, when we were young we were active and with this we’re trying to work out what we can and what we can’t do and what he can eat and try to help him.

Parents reflected on the negative effects of comorbid conditions on their children’s severe obesity and feelings of hopelessness about future reductions of this level, even when engaging in making health related behaviour change, such as Carly (P3):

He’s got an absent posterior bright spot which is the bit that controls your hypothalamus. So it’s not his fault. The messages aren’t quite – they’re misfiring to tell him that he’s either full or had enough. It slows his metabolism down so even though we do such a restricted diet and we do a lot of exercise, it’s just he’s always going to be carrying a little bit extra.

#### Difficulties with medical professionals in seeking support for reductions in child level of obesity

Parents all described seeking health-related behaviour advice from differing medical doctors (often termed “General Practitioners”, or “GPs”, in Commonwealth countries such as Australia) and specialists to address their child/adolescent’s severe obesity, and advice specific for their child/adolescent’s comorbid medical diagnoses. Parents described repeated efforts to get help, but experienced minimisation of their children’s health problems by professionals, professionals blocking access to specialist services, feelings of frustration with negative and unhelpful attitudes of medical professionals, perceptions that specialist advice did not help, feeling judged by specialists who assumed parents were unwilling to follow their advice, and loss of confidence in specialist advice.

All families described repeatedly seeking help from primary care medical professionals (GPs) when seeking support specific to their child/adolescent’s severe obesity, such as Robyn (P5) “[Child names] been through quite a bit already, with regards to weight, so…Well, he’s been to – so, lots of GP visits, because we’re concerned about it”. Parents often described their worries were minimised by these professionals, like Delia (P8):

Over the years I’ve brought it up so many times with different GPs about her weight, and they all just kind of fob it off, like “Oh, she’s not that bad”. I’m like “Well, it’s not normal for a child to be this size”.

Parents also described being actively blocked by GPs in seeking additional help from other specialist services: “That’s why I’m thinking “What’s wrong with this kid?”. That’s why I said I wanted to go and see an endocrinologist, but this GP has just said no, just do the blood tests”. Delia (P8).

Many families were or had engaged with medical specialists, such as psychologists, paediatricians or dieticians, to address their child/adolescent’s severe obesity. Parents often described feeling frustrated with the negative and unhelpful attitudes of these professionals, like Carly (P3),

He [child] was probably 18 months old or maybe it was a bit earlier when I got the diagnosis. I thought there was something wrong – he [Paediatrician] said to me, “There’s no name for it. It is what it is. It’s just a birth defect and there’s nothing you can really do, maybe a bit of physio.” So, he [the Paediatrician] was a bit of a douche.

Parents described that medical specialists were unable to provide guidance that resulted in any change in health behaviours or reduction in the level of obesity in their child, as suggestions had been tried but had failed, such as Jane (P13):

They gave me a food diary, how much portion I should give to [Child name] and just the cycle. But I wasn’t able to get back to the dietician this year. It’s not helpful because what he eats is still the same. So there’s no variety or whatever and it’s just morning tea, lunch or whatever. It’s still the same. There’s nothing…There’s no change at all.

Some parents described feeling that specialists assumed parents were unwilling to follow their advice, leading to frustration. Such judgment was often expressed as leading to a loss of confidence in advice from medical professionals, such as Mel (P4): “When you see a nutritionist or a dietician they don’t seem to actually understand about autism, that it’s not just being a brat, it’s an actual - it’s not going to go away”.

## Discussion

The purpose of this study was to better understand the experiences and challenges of parents who were not or had chosen not to engage in a clinical tertiary obesity intervention program for children/adolescents in facilitating a healthy lifestyle for their child with severe obesity. Taken together, our findings advance our understanding of the familial challenges associated with supporting healthy lifestyles for children/adolescents with severe obesity by highlighting several broad difficulties. More specifically, these results highlight that the way parents view “weight loss”—or their perceived inability to achieve it—for themselves or their child/adolescent was a key factor limiting their capacity to support health behaviours. In turn, these negative beliefs appeared to reinforce parents’ beliefs about the inevitability of continued severe obesity for their child/adolescent. In addition to these challenges, parents also described difficulties getting support from medical professionals, suggesting that this group may be isolated and missing key allies or supporters of change.

Some of the barriers to supporting a child/adolescent with severe obesity living a healthy lifestyle described by the parents with obesity in this study are echoed in the obesity and weight management literature in adults —factors such as previous failed attempts [36], feelings that weight loss was out of their control [37], motivation problems [38] and body image and self-esteem issues [38]. However, we consider our findings important insofar as they demonstrate that parents with obesity may view health-related behaviour change—for themselves and also their child/adolescent—through their own lifelong-obesity narrative lens. This narrative, as well as other parental experiences reported in this study (e.g., inevitability), may be due partly to the notion that parents have learned or adopted a *fixed* mindset (i.e. that they believe an attribute to be unchangeable) [39] about their ability to achieve any weight loss, which is underpinned by a perceived lifetime of cyclical failed attempts of achieving weight loss. Parents’ perception that their child/adolescents obesity was beyond parents control has been discussed previously [6], although our findings are novel in that they highlight this perception among parents of children with severe obesity.

Parents in our study also commonly felt frustrated that they had failed to promote weight loss in their child/adolescent, a feeling of failure that has been reported in other studies [14, 26]. It is possible that parents’ feelings of frustration at repeated failed attempts of child/adolescent weight loss and internalised weight biases [40], may activate their own lifelong obesity narrative, and may also explain why parents with obesity in this study perceived obesity in their child/adolescent as hopeless and *inevitable*. This inevitability belief has been found in studies demonstrating that adults with obesity more strongly endorse the notion that obesity is inherited—a belief that has been shown to be associated with lower physical activity levels and poorer food choices [41]. Thus, our results highlight an important implicit barrier that appears to negatively frame parents’ beliefs about their everyday ability to support their child/adolescent with severe obesity in living a healthy lifestyle. Therefore, in seeking to better understand and address possible mindset-related barriers, it may be valuable to examine factors associated with changing mindsets—from a fixed to a more growth-focused perspective—in a health context [42].

The results of this study also highlight several problematic barriers associated with seeking medical support. First, our results highlight differences to other studies in that parents of children/adolescents with severe obesity often *avoid* seeking medical help due to being blamed for the level of obesity in their child [26] - this did not appear to be the majority experience for parents in our study. Participants in the present study did express a desire to seek help, but also reported being blocked from seeking further specialist treatment by primary medical care doctors (GPs). Authors of other studies have also reported similar issues with healthcare professionals, where parents specifically sought referrals to specialists for their child/adolescent with severe obesity as the desired outcome from primary care doctors due to the belief that these doctors did not have the skills to manage treatment of the obesity level [26]. What is also problematic is that even once parents received a referral to a specialist medical practitioner, these specialists were perceived, in some cases, to be judgemental of parents, and the help and guidance they received was often ineffective in changing their child/adolescents’ level of obesity. This judgement from health professionals may stem from professionals’ weight bias or stigma against individuals with obesity [40]. Weight stigma refers to the idea that individuals devalue people because of their weight [40]. Other studies have supported the notion of implicit weight biases held by health professionals working within paediatric weight treatment programs [9]. In addition, other studies have shown that health care specialists may express bias towards parents with obesity of children/adolescents with obesity, rather than the child/adolescent themselves, and blame parents for poor lifestyle adherence [26, 43] or infer parenting failure due to the parent’s own level of obesity [9, 43]. Our results highlight that parents are experiencing significant difficulties in seeking support from medical professionals, creating an additional barrier in supporting their child/adolescent with severe obesity.

### Clinical implications

Our results prompt several important clinical implications. Firstly, clinical intervention teams for severe child/adolescent obesity are encouraged to consider and investigate the history of parental levels of obesity and health beliefs prior to a child/adolescent and family engaging in a program. This should not preclude parents of families that have longstanding obesity histories; rather, parents may benefit from support around their own—and their child/adolescent’s—obesity narratives and mindsets prior to engaging in a treatment program. This suggestion is in line with literature in treatment of obesity in adults, where psychological barriers such as maladaptive eating patterns and motivation, emotional regulation problems and maladaptive/unhelpful thought patterns are best addressed prior to focussing on treatment for weight loss [44]. Secondly, it appears that one of the factors that complicates help seeking is that seeking help itself was made difficult by medical professionals. Parents perceived difficulties in accessing primary care and support from medical professionals, and when they did receive professional support, they often felt negatively judged. These parental perceptions align with literature in primary medical care settings where GP’s identified deficits in their own skills to treat and support parents of a child/adolescent with severe obesity [45]. These parental perceptions also suggest that medical professionals working with severe obesity should be encouraged to consider and address their own weight biases, and where identified, seek appropriate professional education or training. These results highlight the fact that severe levels of obesity are more complicated, and comorbidities further complicate a difficult situation. Therefore, parents may require more targeted specialised help, and specialists may need upskilling and education to be able to address and support these parents to deliver and encourage families to engage in meaningful long-term changes. Additionally, our results highlight implications for the design and delivery of programs, as they provide insight into the complex experiences and histories that are important to consider alongside program structure and delivery issues.

### Strengths and opportunities for future research

This study differs from existing reports in that the parental opinions are reflective of differing socioeconomic backgrounds (i.e. participants SEIFA rankings ranged from 1 to 10), and importantly, while many studies have been limited to either very young children (2–5 years), young children (6–12 years), or adolescents (13 years plus), the parents in this study had children of varying ages. Further, this population of families—characterised by children/adolescents who have severe obesity but are not engaged in treatment—are often considered hard to access and are, as a result, not as well understood in terms of experiences and challenges compared to the more ‘general’ populations with (less severe) levels of overweight or obesity. Additionally, as people with obesity are known to be subject to stigma due to their weight [10, 46], it may be that parents with varying levels of obesity are not always willing to participate in research for fear of judgement from researchers [10, 14, 15] or medical professionals [26]—sourcing opinions form this cohort is important in designing future interventions. This study also directly ties the obesity status of parents, and distorted cognitive beliefs about weight loss, as a specific barrier that could be addressed in the design of future interventions for child/adolescents with severe obesity. There are several aspects of this study that could be explored in future research. Firstly, participants resided in the metropolitan area of Perth and therefore its unknown if families in outer areas, or regionally, have experienced similar or additional difficulties. The rise of the use of online platforms may enable future research to reach more families. Secondly, the role of parents internalised weight biases and experiences of weight stigma which is often reinforced by interactions with healthcare and society, should be investigated on a larger scale. Lastly, whilst the lead researcher invited families from a large recruitment pool (*N* = 103), only 13 families consented to participate. Further, both parents were invited to attend interviews, only one parent attended per family, and most commonly mothers. Future research could investigate in a larger sample, whether fathers or whole family perspectives (both fathers and mothers or primary caregivers) may present different perspectives on challenges in supporting their child/adolescent with severe obesity.

## Conclusion

For some parents, negative underlying fixed cognitive views of weight loss may prohibit active and committed support of their child/adolescent’s journey of weight loss. Adding to these cognitive orientations, parents in this study reported difficulties, negative judgments, and poor advice from medical professionals in terms of their child/adolescent’s level of obesity. Within the literature there is a negative and stigmatizing narrative that parents ignore the gravity of their child/adolescent’s level of obesity or ignore the advice from specialists. Importantly, this study offers an alternative perspective—that medical professionals may be contributing to barriers and parents’ feelings of internalised hopelessness about their child/adolescent’s level of obesity. Consideration of this perspective is crucial, and clinically relevant to child and adolescent treatment programs for severe obesity.

## Data Availability

Due to the nature of this research, participants of this study did not agree for their data to be shared publicly (as participants individual privacy could be compromised), so supporting data is not available. Please contact liz.saunders@research.uwa.edu.au for further details.
